# Light quality regulates flowering in *FvFT1/FvTFL1* dependent manner in the woodland strawberry *Fragaria vesca*

**DOI:** 10.3389/fpls.2014.00271

**Published:** 2014-06-11

**Authors:** Marja Rantanen, Takeshi Kurokura, Katriina Mouhu, Paulo Pinho, Eino Tetri, Liisa Halonen, Pauliina Palonen, Paula Elomaa, Timo Hytönen

**Affiliations:** ^1^Department of Agricultural Sciences, University of HelsinkiHelsinki, Finland; ^2^Department of Electrical Engineering and Automation, Aalto UniversityEspoo, Finland; ^3^Department of Biosciences, University of HelsinkiHelsinki, Finland

**Keywords:** flowering, Fragaria, FT, light spectrum, perennial, SOC1, strawberry, TFL1

## Abstract

Control of flowering in the perennial model, the woodland strawberry (*Fragaria vesca* L.), involves distinct molecular mechanisms that result in contrasting photoperiodic flowering responses and growth cycles in different accessions. The *F. vesca* homolog of TERMINAL FLOWER1 (FvTFL1) functions as a key floral repressor that causes short-day (SD) requirement of flowering and seasonal flowering habit in the SD strawberry. In contrast, perpetual flowering *F. vesca* accessions lacking functional FvTFL1 show FLOWERING LOCUS T (FvFT1)-dependent early flowering specifically under long-days (LD). We show here that the end-of-day far-red (FR) and blue (B) light activate the expression of *FvFT1* and the *F. vesca* homolog of *SUPPRESSOR OF THE OVEREXPRESSION OF CONSTANS* (*FvSOC1*) in both SD and LD strawberries, whereas low expression levels are detected in red (R) and SD treatments. By using transgenic lines, we demonstrate that *FvFT1* advances flowering under FR and B treatments compared to R and SD treatments in the LD strawberry, and that *FvSOC1* is specifically needed for the B light response. In the SD strawberry, flowering responses to these light quality treatments are reversed due to up-regulation of the floral repressor *FvTFL1* in parallel with *FvFT1* and *FvSOC1*. Our data highlights the central role of FvFT1 in the light quality dependent flower induction in the LD strawberry and demonstrates that FvTFL1 reverses not only photoperiodic requirements but also light quality effects on flower induction in the SD strawberry.

## Introduction

Plants monitor light intensity, duration, spectrum, and direction to adjust their growth and development. Photoperiod changes regularly throughout the year, and therefore, many plants rely on photoperiodic signals to control important phase transitions including flower induction. Furthermore, specific regions of the light spectrum have different effects on flowering. For example, far-red (FR) light, which is enriched under canopy, causes early flowering in many species (Brown and Klein, 1971; Johnson et al., 1994; Cerdán and Chory, 2003; Wollenberg et al., 2008). Plants sense changes in light by using photoreceptors. Phytochromes (Phy) are the only photoreceptors known to mediate photomorphogenic red (R) and FR signals (Takano et al., 2009; Strasser et al., 2010). In Arabidopsis [*Arabidopsis thaliana* (L.) Heynh.], PhyA promotes, whereas PhyB, D, and E repress flowering (Johnson et al., 1994; Reed et al., 1994; Devlin et al., 1998, 1999; Hu et al., 2013). In addition, cryptochrome (Cry) and light oxygen voltage (LOV) receptors mediate the effect of blue (B) light to control flowering (Guo et al., 1998; Mockler et al., 1999, 2003; Sawa et al., 2007).

Genes that are regulating photoperiodic flowering are conserved between annual short-day (SD) and long-day (LD) model species rice (*Oryza sativa* L.) and Arabidopsis, respectively, although their mode of action may differ (Hayama et al., 2003; Tsuji et al., 2011; Brambilla and Fornara, 2013). Photoperiodic flowering is explained by the external coincidence model. In Arabidopsis, *CONSTANS (CO)* mRNA is expressed rhythmically with a peak around the dusk under LD conditions and during the night under SD (Suarez-Lopez et al., 2001). Since CO is unstable in darkness, the protein accumulates only under LD when light coincides with the *CO* mRNA expression in the afternoon (Valverde et al., 2004). CO activates *FLOWERING LOCUS T (FT)* in the leaf phloem, and the FT protein moves into the shoot apical meristem (SAM) (Corbesier et al., 2007; Jaeger and Wigge, 2007; Tamaki et al., 2007). At the SAM, FT binds with the bZIP transcription factor FD, and this complex up-regulates the gene expression of MADS transcription factor APETALA1 (AP1) to induce flowering (Abe et al., 2005; Wigge et al., 2005). Also in SD plant rice, photoperiodic flowering occurs through external coincidence. Rice *CO* homolog, *Heading date 1* (*Hd1*), has similar expression pattern with *CO*. However, Hd1 activates *FT* homolog *Heading date 3a* (*Hd3a*) only under SD through an unknown mechanism which includes the action of PhyB (Kojima et al., 2002; Cremer and Coupland, 2003; Ishikawa et al., 2011; Tsuji et al., 2011). Furthermore, Hd3a forms a florigen activator complex with 14-3-3 proteins and OsFD1 to activate OsMADS15, a homolog of AP1, to induce flowering (Taoka et al., 2011).

Light quality affects the transcription of *CO* and *FT* and the stability of CO protein via different photoreceptors. Yanovsky and Kay (2002) showed that both Cry2 and PhyA are involved in the normal activation of *FT* mRNA expression in the photoperiodic flowering. These photoreceptors play partially redundant role to mediate B light promotion of flowering together with Cry1, although the main role of PhyA is to mediate the FR signal (Mockler et al., 2003). These photoreceptors are involved in the stabilization of CO protein, whereas PhyB promotes the degradation of CO in R light (Valverde et al., 2004). B light activated LOV-receptor, FLAVIN BINDING, KELCH REPEAT, F-BOX1 (FKF1), stabilizes CO protein specifically in the afternoon when CO promotes the expression of *FT*. FKF1 is also directly involved in the transcriptional regulation of both *CO* and *FT* (Imaizumi et al., 2003; Sawa et al., 2007; Song et al., 2012). PhyB and other light stable phytochromes have additional roles in shade avoidance conditions. Low R/FR ratio inactivates PhyB, which leads to the activation of the photoperiodic flowering pathway. An additional regulator, PHYTOCHROME AND FLOWERING TIME1 (PFT1) promotes the expression of *CO* and *FT* by repressing Phy signaling (Cerdán and Chory, 2003; Wollenberg et al., 2008).

In perennials, the molecular level studies on the light regulation of flowering have focused on *Populus* and on the woodland strawberry, *Fragaria vesca* L. that represents the model species for the economically important Rosaceae family (Böhlenius et al., 2006; Hsu et al., 2011; Koskela et al., 2012; Mouhu et al., 2013). In *F. vesca*, both seasonal flowering and perpetual flowering accessions with contrasting photoperiodic responses exist (Brown and Wareing, 1965). Seasonal flowering accessions are SD plants (Heide and Sønsteby, 2007). In perpetual flowering *F. vesca*, however, LD advances flower induction, but plants eventually flower also under SD (Sønsteby and Heide, 2008; Mouhu et al., 2009; Koskela et al., 2012). A strong floral repressor, *F. vesca* homolog of *TERMINAL FLOWER1* (*FvTFL1*) has been shown to control seasonal flowering, whereas perpetual flowering accessions have non-functional *FvTFL1* alleles with a 2 base pair deletion in the first exon (Iwata et al., 2012; Koskela et al., 2012). Also in the cultivated strawberry (*Fragaria* × *ananassa* Duch.), both seasonal and perpetual flowering cultivars with similar environmental responses are known (Heide, 1977; Sønsteby and Heide, 2007; Bradford et al., 2010; Kurokura et al., 2013).

Molecular analyses in *F. vesca* have revealed that homologs of FT and SUPPRESSOR OF THE OVER-EXPRESSION OF CONSTANS1 (FvFT1 and FvSOC1) may mediate the photoperiodic signal to control flowering through *FvTFL1*. These genes seem to form a linear pathway in which FvFT1 promotes the expression *FvSOC1*, which leads to increased *FvTFL1* mRNA levels (Mouhu et al., 2013). Since FvTFL1 is a strong floral repressor, the activation of this pathway under LD maintains the plants at the vegetative stage (Koskela et al., 2012). Under SD in autumn, however, the expression of *FvFT1* and *FvSOC1* decrease leading to the down-regulation of *FvTFL1*, and consequently, the up-regulation of *FvAP1* occurs in the shoot apex in parallel with the initiation of floral development. The growth cycle continues in the next spring when determinate inflorescences emerge and produce fruits. Flowering and fruiting overlap with the next yearly growth cycle which begins with the growth of new vegetative axillary shoots with high *FvSOC1* and *FvTFL1* expression level in the spring. In perpetual flowering accession Hawaii-4 (H4), however, the lack of functional FvTFL1 reverses the photoperiodic flowering response, and both FvFT1 and FvSOC1 act as floral activators (Koskela et al., 2012; Mouhu et al., 2013).

Vince-Prue and Guttridge (1973) showed that the end-of-day FR light treatment prevents flower induction in the cultivated strawberry, whereas R light has an opposite effect. To understand strawberry responses to the light quality at the molecular level, we carried out end-of-day treatments with R, FR and B light in the *F. vesca*. We report strong activation of *FvFT1* by FR light, weaker activation by B light, and almost no expression under R light. Using transgenic lines, we show evidence that FvFT1 mediates the promotion of flowering under FR and B light treatments in the perpetual flowering accession H4, which is lacking functional FvTFL1. In the seasonal flowering accession, however, high *FvFT1* expression correlates with high *FvTFL1* mRNA levels, and flowering responses to different light qualities are reversed.

## Materials and methods

### Plant material and growing conditions

Seedlings of seasonal flowering SD accession of the woodland strawberry (*Fragaria vesca* L.) and perpetual flowering LD accession H4 (Accession numbers PI551792 and PI551572, respectively; National Clonal Germplasm Repository, Corvallis, USA) were used. Seedlings were raised in a greenhouse under non-inductive photoperiod (12 or 18 h for H4 and SD *F. vesca*, respectively) at 18 ± 1°C (first experiment) or at 22 ± 1°C. High pressure sodium (HPS) lamps (Airam 400W, Kerava, Finland) were used to supplement natural light with the intensity of 150 μmol m^−2^s^−1^. In SD conditions, darkening curtains were used to exclude any light during the 12 h night. After rooting, seedlings were transplanted to 8 × 8 cm pots. Fertilized peat supplemented with 25% (v/v) of vermiculite (Ø2 mm) was used as a growing media. Plants were fertilized with liquid fertilizer biweekly.

Previously reported *FvFT1* and *FvSOC1* RNAi lines in H4 background (Koskela et al., 2012; Mouhu et al., 2013), and *FvFT1* over-expression lines produced in this work (see below), were analyzed. All transgenic plants were propagated from seeds originating from the self-pollination of the primary transgenic lines. Seeds were germinated on moisturized filter paper on petri dishes at room temperature for 5 days when the primary root was emerged. Since both RNAi and over-expression vectors, pK7GWIWG2(II) and p7WG2D (Karimi et al., 2002), contain green fluorescent protein (GFP) as a selectable marker, we observed GFP signal in the primary roots under the fluorescence microscope (Leica MZ FL3, Leice Microsystems, Wetzlar, Germany) and transferred GFP-positive seeds onto the soil. Transgenic seedlings were raised under SD conditions in greenhouse until the light treatments started. *FvFT1* over-expression lines were moved to the light treatments immediately after germination in order to avoid flower induction before the treatments. Wild type control plants were raised following the same procedure with transgenic seedlings.

### Lighting treatments

Incandescent lamps (INC; R/FR = 0.95; Philips 60W) and light emitting diodes (LED) were used for the end-of-day lighting treatments. R, FR and B LED lighting systems were built up using deep-red (LZ1-10R205; LEDEngin Inc, San Jose, USA), far-red (L735-66-60; Epitex Inc., Kyoto, Japan) and royal-blue (Z-Power D32282; Seoul Semiconductor Co. Ltd., Ansan-city, Korea) high-power LED components with measured peak wavelength emissions at 655, 740, and 455 nm, respectively. These lighting systems were used in the experiments that did not include transgenic lines. However, in the experiments with transgenic lines, Philips Green Power LED research modules (deep R, FR, and B; Philips, Amsterdam, The Netherlands) were used.

Young seedlings were subjected to the end-of-day light quality treatments in a greenhouse rooms equipped with darkening curtains during the winter season (November-March). The developmental stage of the seedlings in the beginning of the treatments is indicated in the figure legends. Plants were illuminated daily for 12 h with 150 μmol m^−2^s^−1^ of HPS light. After 12-h HPS illumination, the plants were subjected to low intensity (8–15 μmol m^−2^s^−1^, as indicated in the figure legends) end-of-day R, FR, B or incandescent light (INC) treatments for 6 h. In addition, 12-h SD was used as a control. Natural light was excluded by using darkening curtains when HPS lamps were turned off. Temperature during the treatments was 18 or 22 ± 1°C (indicated in the figure legends). After the treatment period of 5–8 weeks (as indicated in the figure legends), the plants were transferred to standard LD growing conditions, 18 h of HPS illumination (150 μmol m^−2^s^−1^) at 18 ± 1°C, for flowering observations.

### Growth observations

Flowering time observations were carried out 2–3 times per week to record the date of first open flower. In H4, flowering time was also observed by counting the number of leaves in the primary leaf rosette before the terminal inflorescence.

### Genetic transformation

*FvFT1* was amplified by using primers 5′- aaaaagcaggctGGATCAATATGCCTAGGGACAGG-3′ and 5′- agaaagctgggtAAAGGGTTTACGATGATCTTCTC-3′ (lower case letters indicate the binding site for the Gateway adapter primers), and the resulting fragment was introduced in the p7WG2D over-expression vector (Karimi et al., 2002), which includes GFP as a selectable marker, using Gateway® technology with Clonase™ II (Invitrogen, Carlsbad, USA). This construct was electroporated to the *Agrobacterium tumefaciens* strain GV3101 and transformed to the *F. vesca* accession H4 as described earlier (Oosumi et al., 2006).

### RNA extraction, cDNA synthesis, and real-time PCR

Leaf and/or shoot apex samples were collected for gene expression analyses during lighting treatments in the time points indicated in the figures and figure legends. For the leaf samples, middle leaflets of youngest fully opened leaves, and for shoot apex samples, ~1 mm pieces containing SAM and youngest leaf initials were pooled from several plants. Three biological replicates were collected for each sample. RNA extraction was done by using pine tree method (Monte and Somerville, 2002). For cDNA synthesis (Superscript III reverse transcriptase, Invitrogen) 1 μg of total RNA was used. Real time PCR reactions were performed using SYBR Green Master Mix (Roche, Basel, Switzerland) and 3 μM primer mix (F+R) by using LightCycler 480-instrument (Roche). Real time PCR program is presented in Supplementary Figure 1. Three biological and three technical replicates were analyzed in each experiment. Relative expression of selected genes was measured by ΔΔCt method with stable *FvMSI1* as a normalization gene (Supplementary Figure 2). Real time PCR primers are listed in Supplementary Table1. Primer efficiencies were close to 2 for all primer pairs.

### Statistical analyses

Flowering time results were subjected to the One-Way or Two-Way analysis of variance (ANOVA) using general linear model (GLM-procedure, SAS 9.3 Software, SAS Institute Inc., Cary, USA). Pairwise comparisons were carried out using Tukey's test (α = 0.05 or 0.01 in one-way and two-way ANOVA, respectively).

## Results

### End-of-day FR, but not R light promotes flowering in the *F. vesca* accession H4

**Figure 1:**
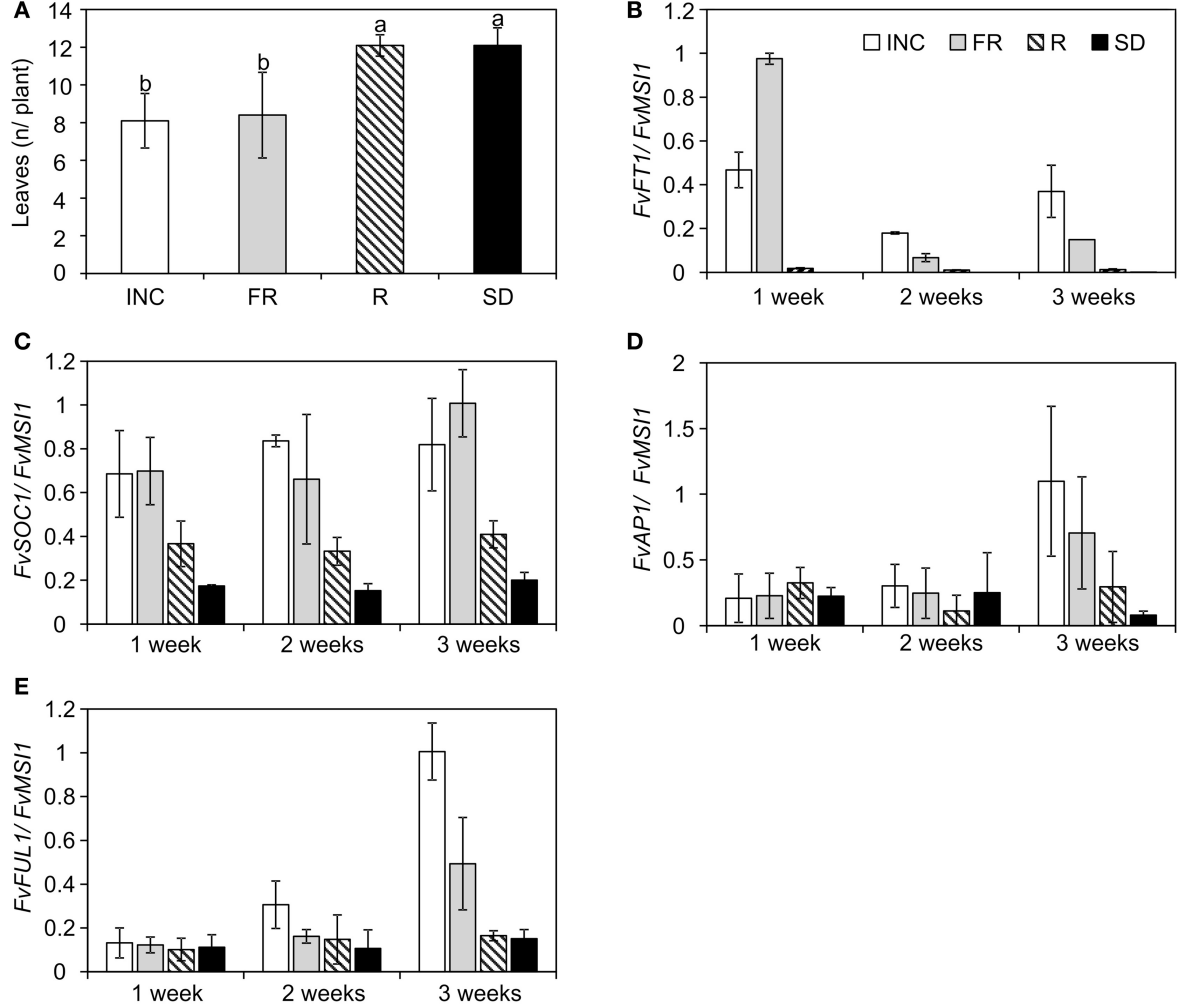
**Flowering time and the expression of flowering time genes under the end-of-day light quality treatments in the perpetual flowering *F. vesca* accession Hawaii-4 (H4). (A)** Flowering time of the seedlings of H4 indicated as the number of leaves in the primary leaf rosette before the terminal inflorescence (*n* = 8–11). Flowering results were subjected to One-Way ANOVA (*t*-test), *p* < 0.001 for the treatment. Different lower-case letters indicate significant differences between the treatments according to Tukey's pairwise test, α = 0.05. **(B)** The expression of *FvFT1* in the leaves of H4 collected in different time points during the treatments. **(C)** The expression of *FvSOC1*, **(D)** *FvAP1*, and **(E)** *FvFUL1* in the shoot apex samples of H4 collected in different time points. Plants with one open leaf were subjected to 12-h short-day (SD) or 12-h SD plus 6-h low intensity (8 μmol m^−2^s^−1^) end-of-day treatment with incandescent, far-red or red light (INC, FR, and R, respectively) at 18°C for 5 weeks. For gene expression data, three biological replicates were analyzed by real-time PCR. Leaf and shoot apex samples were collected 4 h after dawn. All results are mean ± *SD*.

Earlier studies in *F. vesca* have shown that LD advances flowering of the perpetual flowering accession H4 (Mouhu et al., 2009; Koskela et al., 2012). To analyze the effect of light quality on the photoperiodic flower induction in H4, we subjected seedlings to different end-of-day light quality treatments. Plants with one open leaf were exposed to non-inductive SD (12 h day/12 h night), or SD plus low intensity day extension (6 h) of FR, R or incandescent light (INC; R:FR ratio of 0.95; flower inductive LD control) for 5 weeks at 18°C followed by standard LD (18 h) growing conditions (see Materials and methods). In both FR and INC treatments, flower induction was advanced and resulted in terminal inflorescence after about eight leaves in the primary leaf rosette (Figure 1A). In contrast, plants grown under R treatment flowered after 12 leaves similarly to plants grown under SD.

We further tested the effect of light quality at higher temperature of 22°C, since earlier study showed that high temperature enhances photoperiodic responses in the perpetual flowering *F. vesca* accessions (Sønsteby and Heide, 2008). Indeed, we found stronger delay of flowering in R and SD compared to control plants grown under INC than in the experiment carried out at 18°C. Plants grown under SD and R treatments produced 8–9 leaves more than plants under INC treatment and flowered more than a month later (Supplementary Figure 3). We also tested the effect of B light end-of-day treatment and found that B light advanced flowering in H4, but the effect was weaker compared to INC light (Supplementary Figure 3).

### End-of-day FR light induces the expression of the *FvFT1*

Koskela et al. (2012) showed that in H4, *FvFT1* expression correlated with floral induction under LD conditions as well as the expression of putative floral meristem identity genes *FvAP1* and *FvFUL1*. We analyzed the expression of these genes in different end-of-day light quality treatments and found a correlation between the *FvFT1* gene expression level and flowering phenotypes. In the leaves of H4, *FvFT1* was highly expressed in both flowering promoting FR and INC treatments already at 2-leaf stage, 1 week after the beginning of the treatment (Figure 1B). In contrast, we detected very low or no expression in R and SD treatments (Figure 1B), in which the plants flowered late.

In Arabidopsis FT activates the expression of *SOC1* at the SAM (Moon et al., 2005; Yoo et al., 2005), and this regulatory connection was shown to be conserved in the *F. vesca* (Mouhu et al., 2013). We found that *FvSOC1* mRNA levels in the shoot apices partially correlated with the expression of *FvFT1* in the leaves: high expression levels for both genes were detected in INC and FR end-of-day treatments in all tested time points (Figure 1C). However, *FvSOC1* mRNA was detected also in R and SD treatments in contrast to *FvFT1*, but the expression level was lower than in FR and INC treatments. The activation of *FvFT1* and *FvSOC1* in FR and INC treatments was followed by the up-regulation of both *FvAP1*and *FvFUL1* in the shoot apex 3 weeks after the beginning of the treatments but not under SD or R (Figures 1D,E), indicating that flower induction had occurred only in INC and FR treatments at this stage.

At a higher temperature of 22°C, *FvFT1* was also strongly up-regulated in the leaves of H4 under FR light compared to R and SD treatments (Supplementary Figure 3). *FvFT1* expression was detected also in B light, but it was several times lower than under FR treatment.

### Functional role of *FvFT1* and *FvSOC1* in light quality responses

**Figure 2:**
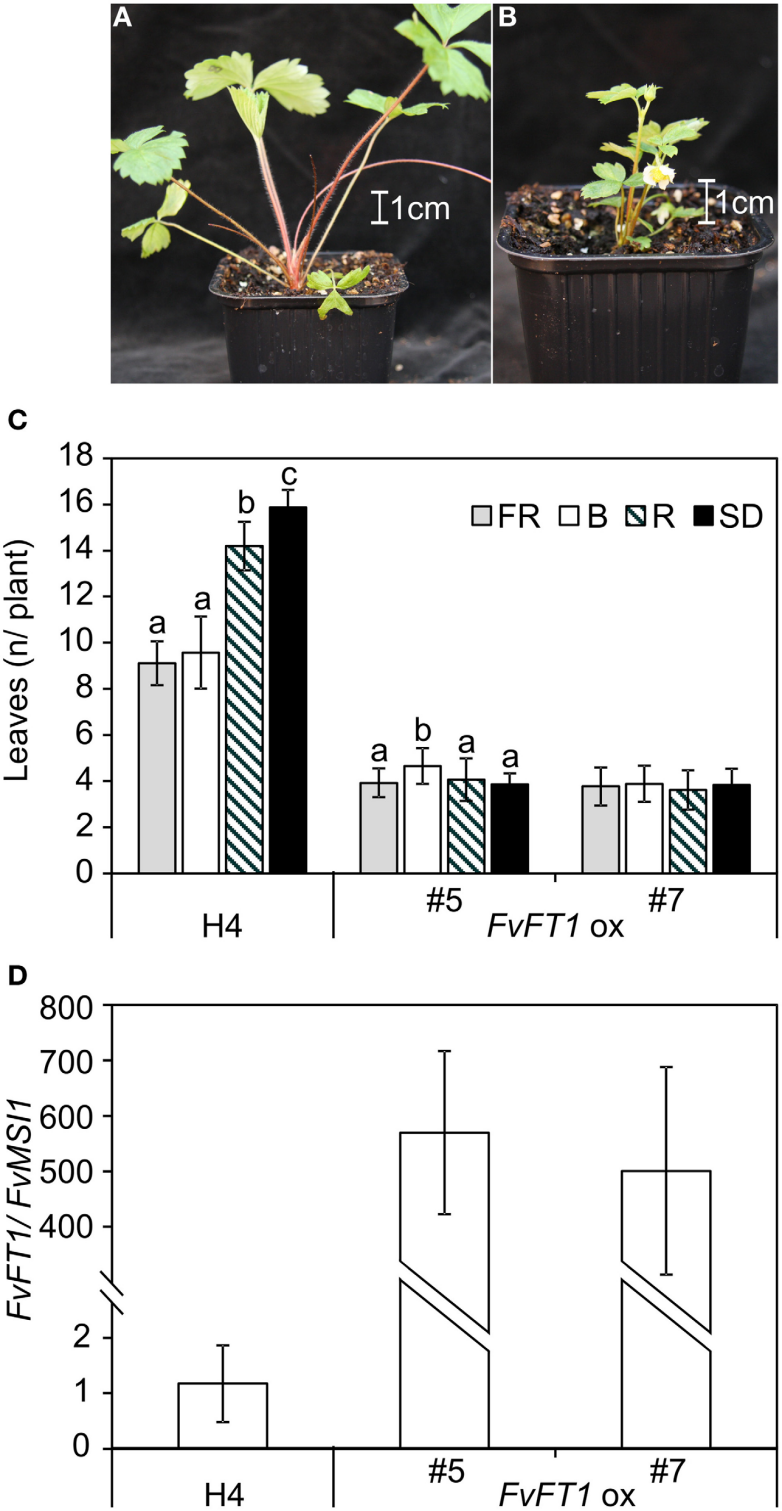
**The overexpression of *FvFT1* causes extreme early flowering in the perpetual flowering *F. vesca* accession Hawaii-4 (H4). (A)** H4 wild type, and **(B)** *35S:FvFT1* line #7 at the age of 8 weeks. **(C)** Flowering time of *FvFT1* overexpression lines in different end-of-day light quality treatments indicated as the number of leaves in the primary leaf rosette before the terminal inflorescence (#5: *n* = 21–31, #7: *n* = 13–18, wild type H4: *n* = 36–38). Flowering results were subjected to Two-Way ANOVA (*p* < 0.001 for each of treatment, genotype, and treatment × genotype interaction). Pairwise comparisons were carried out for every genotype separately using Tukey's test, α = 0.01. Treatments with different letters indicate significant differences within the genotype. **(D)** The expression of *FvFT1* in *35S:FvFT1* plants compared to wild type Hawaii-4 under LD (18 h). T_1_ and H4 seedlings with open cotyledons were subjected to 12-h short-day (SD) or 12-h SD plus 6-h low intensity (15 μmol m^−2^s^−1^) end-of-day treatment with far-red, red or blue light (FR, R, and B, respectively) at 22°C for 8 weeks. For gene expression data, three biological replicates were analyzed by real-time PCR. Leaf samples were collected 16 h after dawn. All results are mean ± *SD*.

**Figure 3:**
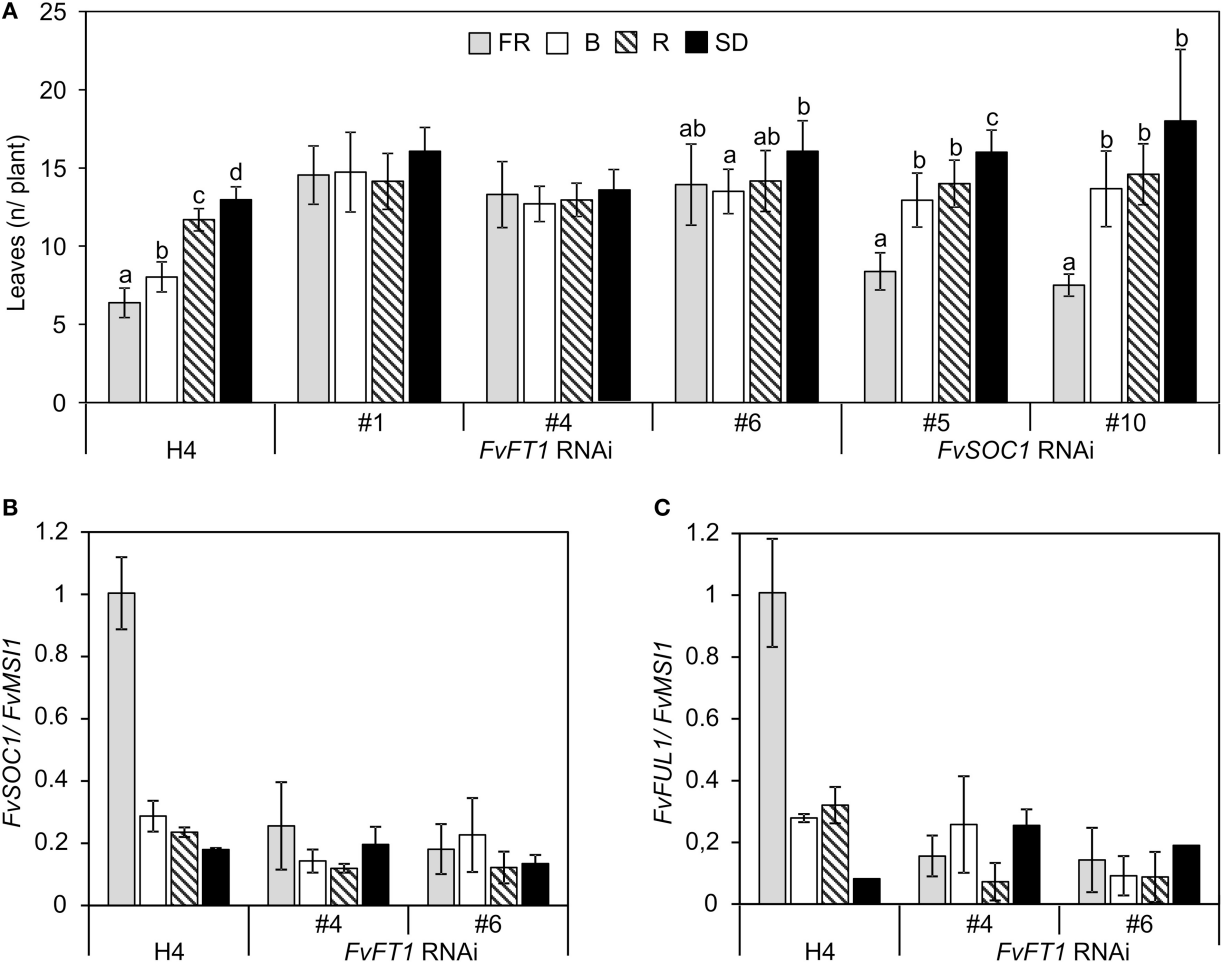
**RNAi silencing of *FvFT1* and *FvSOC1* affects the regulation of flowering by light quality in the perpetual flowering *F. vesca* accession Hawaii-4 (H4). (A)** Flowering time of H4, and *FvFT1* and *FvSOC1* RNAi lines in different end-of-day light quality treatments indicated as the number of leaves in the primary leaf rosette before the terminal inflorescence (*n* = 11–23 for *FvFT1* RNAi lines, *n* = 28–30 for H4, *n* = 13–18 for *FvSOC1* RNAi-5, *n* = 5 for *FvSOC1* RNAi-10). Flowering results were subjected to Two-Way ANOVA (*p* < 0.001 for each treatment, genotype, and treatment × genotype interaction). Pairwise comparisons were carried out for every genotype separately using Tukey's test, α = 0.01. Treatments with different letters indicate significant differences within the genotype. **(B)** Relative expression of *FvSOC1* and **(C)** *FvFUL1* in the shoot apex samples of Hawaii-4 and *FvFT1* RNAi lines. T_1_ and H4 seedlings with 3–4 open leaves were subjected to 12-h short-day (SD) or 12-h SD plus 6-h low intensity (15 μmol m^−2^s^−1^) end-of-day treatment with far-red, red or blue light (FR, R, and B, respectively) at 22°C for 7 weeks. For gene expression data, three biological replicates were analyzed by real-time PCR. Shoot apex samples were collected 4 h after dawn. All results are mean ± *SD*

To confirm the functional role of FvFT1 in different light quality treatments, we used transgenic approach. First, we overexpressed *FvFT1* under cauliflower mosaic virus 35S promoter in H4 background and subjected two independent *FvFT1* overexpression lines to different light quality treatments. The ectopic expression of *FvFT1* led to extremely early flowering compared to non-transgenic H4 (Figures 2A–D). In addition, *FvFT1* overexpression line #7 showed no differences and line #5 minor differences in their responses to various end-of-day light quality treatments while in H4, FR and B light promoted flowering compared to R and SD treatments. Moreover, R light slightly advanced flowering compared to SD in non-transgenic H4 in this experiment. We also tested the responses of three previously reported *FvFT1* RNAi lines in H4 background (Koskela et al., 2012) to the same light quality treatments. In contrast to the wild type H4, FR and B end-of-day treatments did not advance flowering in two *FvFT1* RNAi lines compared to the R and SD treatments, while minor differences between light treatments were observed in the third line (Figure 3A). In this experiment, FR light advanced flowering in non-transgenic H4 more than B light compared to SD or R light treatment, and also R light slightly promoted flowering compared to SD.

Mouhu et al. (2013) showed that *FvSOC1* promotes flowering downstream of *FvFT1*. In line with this result, silencing of *FvFT1* abolished the up-regulation of *FvSOC1* and *FvFUL1* which was observed in wild type H4 under FR light treatment (Figures 3B,C). However, B light treatment did not clearly affect the expression of *FvSOC1* and *FvFUL1* in H4 at this time point due to differences between the observed flowering times and sampling. In this experiment, the plants that received the B light treatment flowered slightly later than those under FR treatment (Figure 3A).

To understand the role of *FvSOC1* in the light quality regulation of flowering, we also studied two independent *FvSOC1* RNAi lines in H4 background (Supplementary Figure 4) under the same light quality treatments. Interestingly, FR treatment accelerated flowering of *FvSOC1* RNAi plants similarly as in the wild type H4 while the effect of B light was absent in the transgenic lines (Figure 3A). This data suggests that FR may induce flowering through FvFT1, independently of FvSOC1, whereas FvSOC1 is needed for early flowering in the end-of-day B light treatment. Taken together, our data on transgenic lines show that FvFT1, in addition to the photoperiodic flowering pathway (Koskela et al., 2012), is the central regulator in the light quality mediated flowering pathway in the perpetual flowering *F. vesca* accession H4. However, according to our data, FvSOC1 may have more specific role in the B light regulation of flowering.

### *FvCO* and *FvFT1* expression peaks do not overlap in light quality treatments

**Figure 4:**
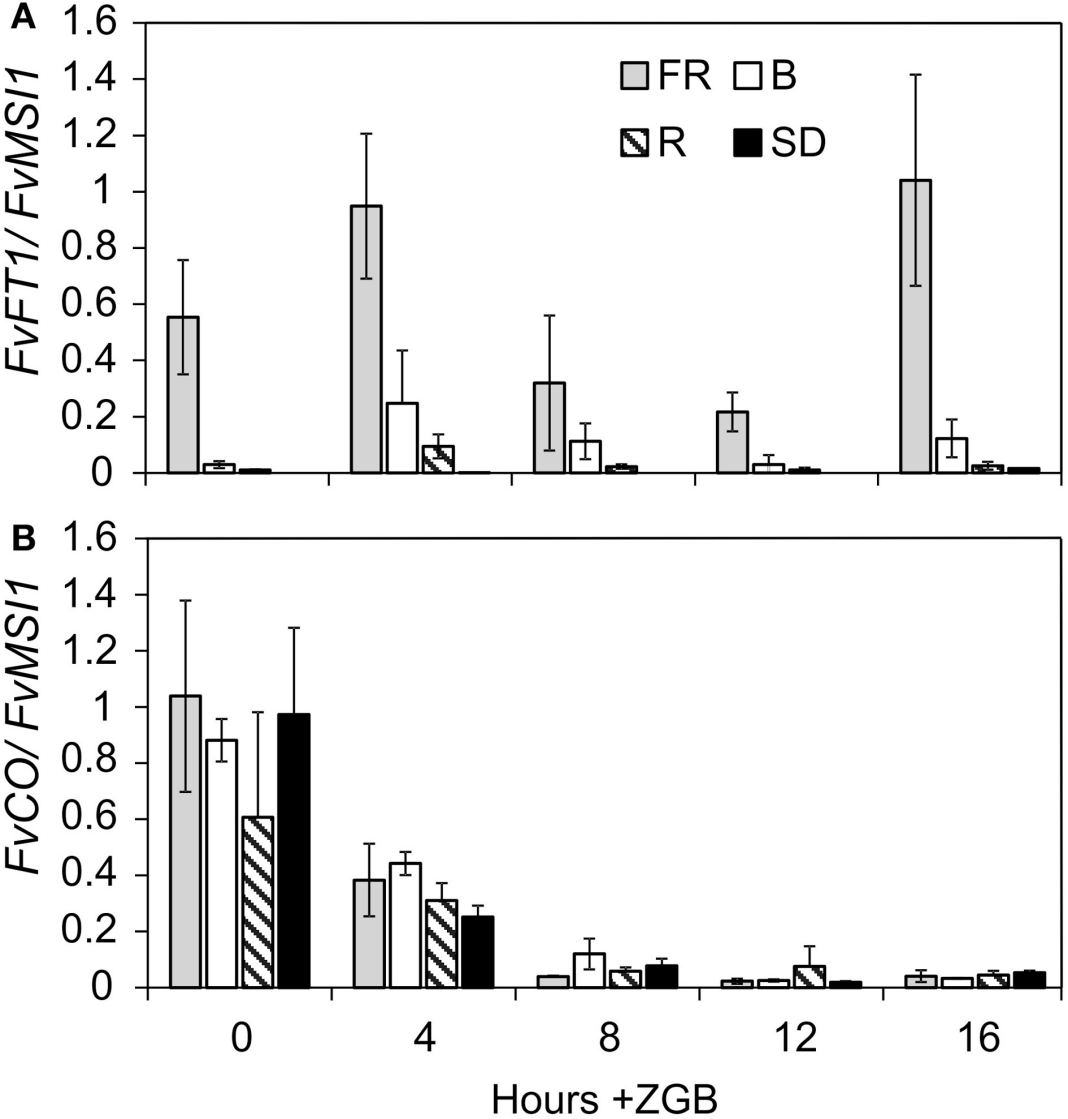
**The effect of the end-of-day light quality treatments on the daytime expression of *FvFT1* and *FvCO* in Hawaii-4. (A)** Relative expression of *FvFT1* and **(B)** *FvCO* in different light quality treatments at 0, 4, 8, 12, and 16 h after lights were switched on (ZGB = zeitgeber time). Values are means of three biological replicates ± *SD*.

In Arabidopsis, the expression of *CO* starts to increase in the afternoon, and CO protein activates *FT* expression in late evening specifically under LD (Suarez-Lopez et al., 2001). In addition, light spectrum affects the expression levels of both genes (Imaizumi et al., 2003; Valverde et al., 2004; Kim et al., 2008; Wollenberg et al., 2008). To get insight into to the putative *CO/FT* module in *F. vesca*, we explored the expression rhythms of *F. vesca CO* and *FT* homologs in different end-of-day light quality treatments. We focused on daytime expression levels, since our earlier data showed that *FvFT1* has a minor expression peak in the morning and another peak in the late evening (Koskela et al., 2012). In the FR treatment, *FvFT1* peaked 4 h after dawn and its expression started to rise again in the evening (Figure 4A). Several times lower expression peaks were detected in B light treatment. Low morning peak (4 h) was observed also in R light treatment, but the expression level of *FvFT1* gene was almost undetectable in the evening.

Several *CO* homologs have been cloned in the cultivated strawberry, and according to phylogenetic analysis, one of those genes, *FrCO*, belongs to the group 1a that includes *CO*, *COL1* and *COL2* in Arabidopsis (Griffiths et al., 2003; Stewart, 2007). We searched for *F. vesca* homologs for *FrCO* from the *F. vesca* genome database (Shulaev et al., 2011; www.rosaceae.org) and found only a single gene with high sequence identity to *FrCO* at the nucleotide and protein level (gene04172-v1.0-hybrid; 97 and 96% identity at the nucleotide and protein level, respectively). We studied the daytime expression rhythm of this gene, which was previously named as *FvCO* (Shulaev et al., 2011), in different light quality treatments. *FvCO* peaked at dawn and its expression decreased along the day (Figure 4B; Supplementary Figure 5). Very low expression level was detected in late evening when the mRNA levels of *FvFT1* were already rising. In addition, light quality treatments did not affect daytime *FvCO* gene expression pattern. These data indicates that if the CO-FT connection exists in *F. vesca*, its mode of action differs from Arabidopsis.

### End-of-day R light down-regulates *FvTFL1* and induces flowering in the SD accession

**Figure 5:**
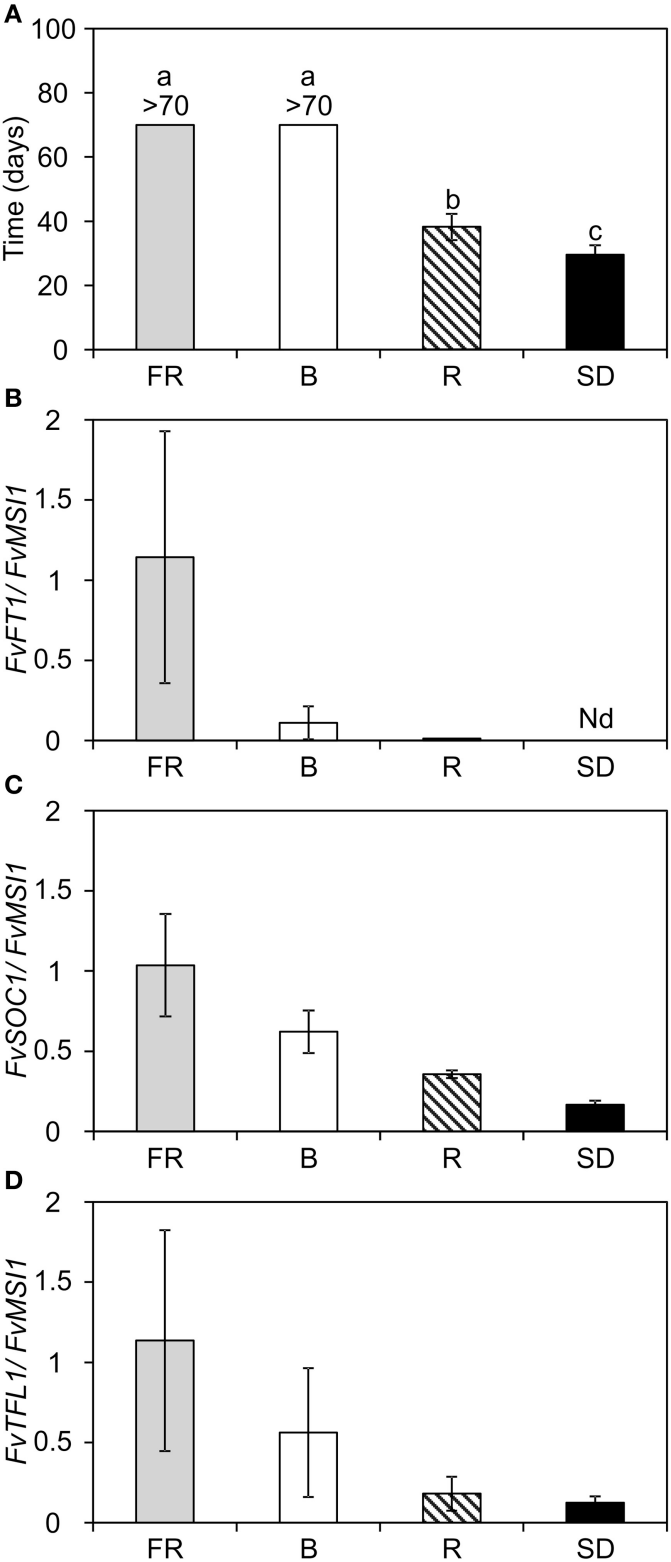
**Flowering time and the expression of flowering time genes in the SD *F. vesca* subjected to end-of-day light quality treatments. (A)** Flowering time of the SD *F. vesca* indicated as days to anthesis after the light quality treatments (*n* = 14). Flowering results were subjected to One-Way ANOVA (*t*-test), *p* < 0.001 for the treatment. Different lower-case letters indicate significant differences between the treatments according to Tukey's pairwise test, α = 0.05. **(B)** The expression of *FvFT1* in the leaf samples, and **(C)** *FvSOC1* and **(D)** *FvTFL1* in the shoot apex samples 4 weeks after the beginning of the light quality treatments. Plants with 5–6 open leaves were subjected to 12-h short-day (SD) or 12-h SD plus 6-h low intensity (15 μmol m^−2^s^−1^) end-of-day treatment with blue, far-red or red light (B, FR, or R, respectively) at 18°C for 8 weeks. For gene expression data, three biological replicates were analyzed by real-time PCR. Shoot apex and leaf samples were collected 4 and 16 h after dawn, respectively. All results are mean ± *SD*. Nd, not detected.

In contrast to the LD flowering H4, seasonal flowering SD *F. vesca* is induced to flower in 12 h SD, whereas 6 h day extension with INC treatment after SD prevents flower induction (Koskela et al., 2012). We subjected the SD *F. vesca* to the similar end-of-day FR, R and B treatments than H4 and found that its flowering responses to different light qualities were reversed compared to H4; R induced, and FR and B prevented flowering (Figure 5A). In R and SD treatments, flowering occurred 38 and 30 days after the end of the 8-week treatment, respectively while the plants under FR and B light treatments stayed vegetative until the end of the experiment.

To understand the observed differences in flowering responses at the molecular level, we analyzed the expression of *FvFT1* in the leaves, and *FvSOC1* and *FvTFL1* mRNA levels in the shoot apex in the SD *F. vesca* 4 weeks after the beginning of the end-of-day light quality treatments. Similarly to H4, in the SD *F. vesca*, *FvFT1* was strongly up-regulated by FR light and some expression was detected in B light, whereas in R and SD treatments, the expression was hardly detected or undetectable, respectively (Figure 5B). Like *FvFT1* in the leaves, *FvSOC1* was up-regulated in the shoot apex of SD *F. vesca* in FR light and expressed only at low level in both R and SD treatments, whereas intermediate levels were observed under B light (Figure 5C). The expression of floral repressor, *FvTFL1*, closely followed that of *FvSOC1* in all light quality treatments (Figure 5D). *FvTFL1* was several-fold down-regulated in floral-inductive SD and R treatments in comparison to FR and B treatments which inhibited flower induction. Taken together, the effect of end-of-day R, FR or B treatment or SD on the expression of *FvFT1* and *FvSOC1* was similar in SD *F. vesca* as in H4, although the flowering responses were opposite. This difference is associated to *FvTFL1* which was co-regulated with *FvFT1* and *FvSOC1* by light. These data suggest that the functional FvTFL1 reverses flowering response to different light qualities in *F. vesca*.

## Discussion

FT has been considered to be a general photoperiodic signaling molecule in both SD and LD plants (Hayama and Coupland, 2004; Lagercrantz, 2009; Pin and Nilsson, 2012). Likewise, in the perpetual flowering *F. vesca* accession H4, *FvFT1* has recently been reported as an LD-induced floral activator which controls the expression of putative floral meristem identity genes *FvAP1* and *FvFUL1* (Koskela et al., 2012). Here we show evidence that *FvFT1* also mediates the effect of light quality to promote flowering in H4. However, in the seasonal flowering SD *F. vesca* with a functional FvTFL1, the effects of the light spectra on flowering are reversed.

### End-of-day FR and B light promote flowering in H4

Flowering of H4 was advanced by the end-of-day treatment of FR or FR-rich incandescent light whereas R light had no effect or very weak effect compared to the SD control. This is a typical response of various LD plants to light quality (Meijer, 1959; Brown and Klein, 1971; Holland and Vince, 1971; Downs and Thomas, 1982; Martinez-Zapater and Somerville, 1990). Since phytochromes are the sole R/FR receptors mediating photomorphogenic and photoperiodic responses (Takano et al., 2009; Strasser et al., 2010), we suggest that these photoreceptors have a major role in the control flowering also in strawberries. However, further studies are needed to confirm which phytochrome(s) mediate the R/FR responses observed in H4.

B light has also been shown to promote flowering in Arabidopsis (Bagnall, 1996; Mockler et al., 2003). We found that the end-of-day B light treatment promoted flowering in H4. However, the effect of B light on flowering was weaker than the effect of the FR or INC light in two out of three experiments reported here. Although these results further suggest that phytochromes are major photoreceptors regulating flowering in *F. vesca*, also B light receptor(s) likely have a role in the control of flowering. In Arabidopsis, cryptochromes and the LOV receptor FKF1 are involved in the B light regulation of flowering (Guo et al., 1998; Imaizumi et al., 2003; Valverde et al., 2004). PhyA, however, can also absorb B light, and it mediates B light signal to control flowering at least in the *cry1cry2* double mutant (Mockler et al., 2003).

### *FvFT1* and *FvSOC1* have distinct roles in the light quality regulation of flowering

In Arabidopsis, light signals mediated by different photoreceptors control the expression of *FT* that promotes flowering (Imaizumi et al., 2003; Mockler et al., 2003; Valverde et al., 2004; Song et al., 2012). Consistent with the up-regulation of *FT* by FR light or low R/FR ratio (Cerdán and Chory, 2003; Mockler et al., 2003; Wollenberg et al., 2008), we found that the end-of-day FR and INC light with R/FR ratio of 0.95 strongly up-regulated *FvFT1* in the leaves of H4, whereas *FvFT1* expression level was very low under R and SD treatments. Also the end-of-day B light treatment somewhat up-regulated *FvFT1* compared to SD. However, consistent with later flowering under B light compared to FR light, several times lower *FvFT1* expression level was detected under B light treatment. The analysis of transgenic lines confirmed the role of FvFT1 in the light quality regulation of flowering in H4. The overexpression of *FvFT1* in H4 caused extreme early flowering independently of the end-of-day light treatment, whereas RNAi silencing of *FvFT1* abolished the FR and B light promotion of flowering. These results indicate that *FvFT1* does not only activate flowering under LD (Koskela et al., 2012) but also controls flowering according to light quality signals perceived by phytochromes and B light receptors.

FT is a positive regulator of *SOC1* in Arabidopsis (Yoo et al., 2005; Torti et al., 2012), and this regulatory link is present also in the *F. vesca* (Mouhu et al., 2013). In this study, we found highest *FvSOC1* expression levels in FR treatment, where the *FvFT1* expression level was also highest. Furthermore, the silencing of *FvFT1* prevented the up-regulation of *FvSOC1* by FR light indicating that FvFT1 mediates at least the FR light regulation of FvSOC1. However, FvFT1 can control flowering independently of FvSOC1 under FR light, since the silencing of FvSOC1 did not affect flowering time under FR light. This is in line with the observation that *FT* and *SOC1* act redundantly to promote flowering under FR enriched light in Arabidopsis (Kim et al., 2008). Although the end-of-day B light treatment advanced flowering compared to SD in non-transgenic H4, this did not occur in *FvSOC1* RNAi lines suggesting that FvSOC1 is needed specifically for the B light promotion of flowering. Taken together, both FvFT1 and FvSOC1 are involved in the control of flowering by the end-of-day B treatment, whereas the promotion of flowering by the FR treatment can occur independently of FvSOC1.

### Functional *FvTFL1* reverses the end-of-day light quality responses in *F. vesca*

Several lines of data support the presence of FvFT1-FvSOC1-FvTFL1 regulatory pathway in the SD *F. vesca*. FvFT1 up-regulates *FvSOC1* in the shoot apex at least in H4, and FvSOC1 activates the expression of *FvTFL1* that encodes a strong floral repressor. Therefore, the photoperiodic flowering response is reversed in the SD *F. vesca* compared to H4 (Koskela et al., 2012; Mouhu et al., 2013). We found that in the SD *F. vesca*, similarly to H4, *FvFT1* and *FvSOC1* gene expression levels were higher under the end-of-day FR and B light treatments compared to R and SD treatments. However, in contrast to H4, SD *F. vesca* was induced to flower under R light and SD treatments while FR and B light inhibited flowering. Taken together, the expression of *FvFT1* and *FvSOC1* correlated negatively with the flower induction in the SD *F. vesca* in all light quality treatments tested in this study as well as in the photoperiodic treatments in previous works (Figure 5; Koskela et al., 2012; Mouhu et al., 2013). We also found that the expression of *FvTFL1* closely followed that of *FvSOC1* in all light quality treatments indicating that the presence of the functional FvTFL1 not only reverses photoperiodic flowering response (Koskela et al., 2012), but also the effect of the end-of-day B light and the phytochrome-mediated R/FR light on flowering. The ortholog of FvTFL1 may control light responses also in the cultivated strawberry, since the SD cultivar of the cultivated strawberry responds to R/FR treatments similarly to the SD *F. vesca* (Vince-Prue and Guttridge, 1973) and the *F. vesca* is one of its ancestors (Hirakawa et al., 2013). Although significant amount of data support the presence of FvFT1-FvSOC1-FvTFL1 pathway in the SD *F. vesca*, functional analysis is needed to confirm whether FvFT1 acts as an anti-florigen in the presence of FvTFL1. Antiflorigens have recently been reported in sugar beet and chrysanthemum (Pin et al., 2010; Higuchi et al., 2013).

### *FvCO* gene expression do not coincide with *FvFT1* mRNA peak

In the *F. vesca*, *FvFT1* mRNA expression peaks in the late evening under LD (Koskela et al., 2012), similarly to *FT* homologs in several other species (Cremer and Coupland, 2003; Böhlenius et al., 2006; Pin et al., 2010). In Arabidopsis, *FT* is induced by CO when *CO* mRNA expression peak coincide with light in the evening under LD (Suarez-Lopez et al., 2001). Our data do not support similar regulation in the *F. vesca*. The Arabidopsis coincidence model would require *FvCO* to peak in the afternoon before *FvFT1*. However, *FvFT1* is highly up-regulated 16h after dawn, when the *FvCO* expression level is low, and *FvCO* mRNA level peaks later toward dawn similarly to Arabidopsis *COL2* and *Populus deltoides CO1* and *CO2*, which have little or no effect on the onset of flowering (Ledger et al., 2001; Hsu et al., 2012).

Both FR and B light increase the expression of *CO* and *FT* in Arabidopsis, whereas lower mRNA levels are observed in R rich light (Imaizumi et al., 2003; Valverde et al., 2004; Kim et al., 2008; Wollenberg et al., 2008). Although light quality treatments strongly affected *FvFT1* mRNA levels in *F. vesca* and caused an additional *FvFT1* expression peak 4 h after dawn, the end-of-day light quality treatments had no effect on *FvCO* daytime expression. Since light quality affects the stability of CO protein in Arabidopsis (Valverde et al., 2004), one possible scenario is that the stabilization of FvCO in the morning up-regulates *FvFT1*. However, further studies are needed to reveal whether FvCO has a role in the regulation of *FvFT1* in different phases of the diurnal cycle in the *F. vesca*.

## Concluding remarks

**Figure 6:**
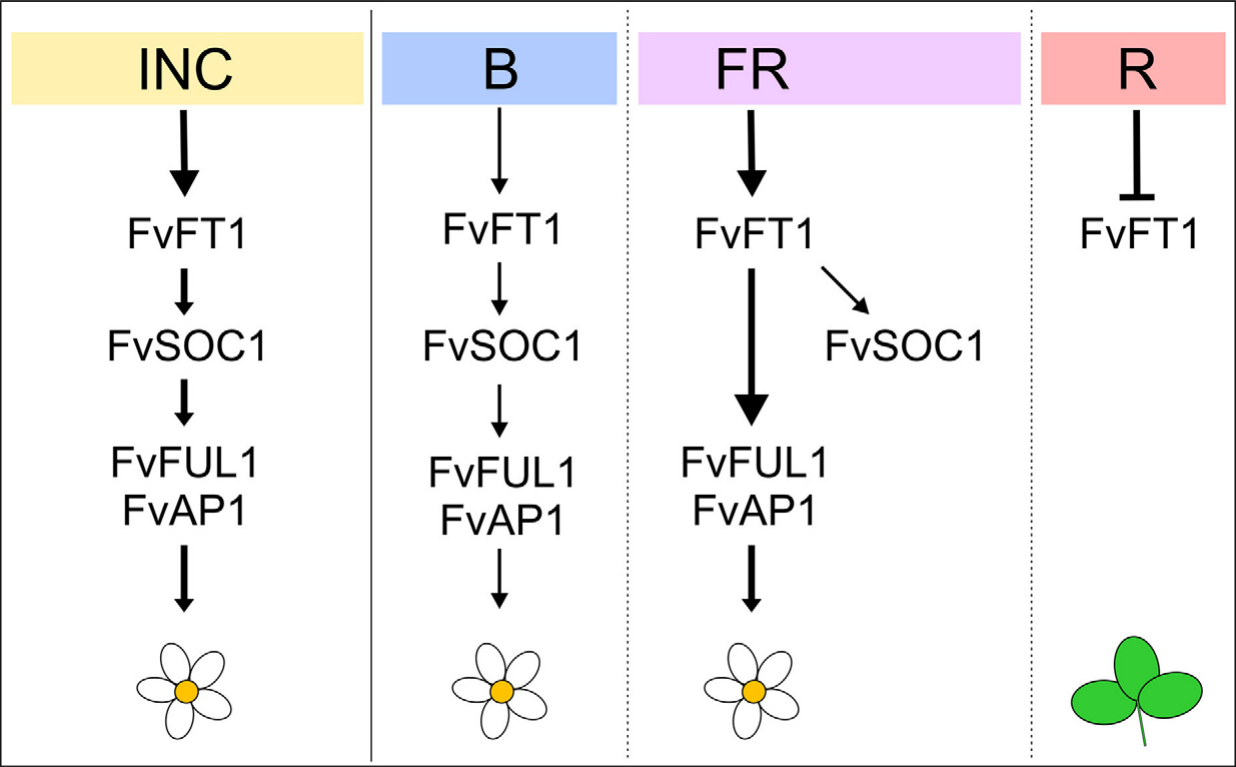
**Model showing the regulation of flowering by light quality in the *F. vesca***. Daylength extension with incandescent light (INC) promotes flowering through the up-regulation of *FvFT1* and *FvSOC1* in the perpetual flowering *F. vesca* accession Hawaii-4 (Mouhu et al., 2013). These genes are essential also for the promotion of flowering by the end-of-day blue (B) light. Under FR light, however, FvFT1 induces flowering independently of FvSOC1, whereas R (red) light down-regulates *FvFT1* and prevents flower induction. In the SD *F. vesca*, FvTFL1, a strong floral repressor that functions downstream of FvFT1 and FvSOC1, reverses the responses to different light qualities. Arrows indicate activation, and bars indicate repression. Thicker line indicates stronger response.

We have shown that both B and R/FR light signals contribute to the regulation of flowering and flowering time genes in *F. vesca*, although phytochrome mediated R/FR signals have stronger effect at least in the end-of-day treatments (Figure 6). In the perpetual flowering LD accession H4, the floral promoter FvFT1 has a central role in flowering responses to different light qualities, whereas FvSOC1 seems to be specifically required for B light mediated activation of flowering. In the SD *F. vesca*, however, the flowering response to different light spectra is reversed, because of the up-regulation of the strong floral repressor FvTFL1 by FvFT1/FvSOC1. These new insights highlight the importance of the regulation of *FvTFL1* and *FvFT1* also in the light quality responses, in addition to photoperiodic flowering, in the perennial Rosaceae model plant *F. vesca* (Koskela et al., 2012). Our result, that flowering of both perpetual (LD) and seasonal (SD) flowering strawberries can be controlled by light quality treatments, may have practical applications in the strawberry cultivation under controlled climate.

## Author contributions

Marja Rantanen, Pauliina Palonen, Paula Elomaa and Timo Hytönen planned the study. Marja Rantanen, Takeshi Kurokura and Katriina Mouhu carried out the experiments and analyzed the data. Paulo Pinho, Eino Tetri and Liisa Halonen designed and Paulo Pinho constructed the LED luminaires. Marja Rantanen and Timo Hytönen wrote the manuscript with input from other authors. All authors read and approved the final manuscript.

## Conflict of interest statement

The authors declare that the research was conducted in the absence of any commercial or financial relationships that could be construed as a potential conflict of interest.

## Supplementary material

Supplementary Table 1 **qPCR primers used in this study**.

Supplementary Figure 1 **qPCR program used in this study**.

Supplementary Figure 2 **Stability of the normalization gene *FvMSI1***.

Supplementary Figure 3 **Flowering time and *FvFT1* expression in Hawaii-4 at 22°C**.

Supplementary Figure 4 **The down-regulation of *FvSOC1* in the RNAi-lines**.

Supplementary Figure 5 **Diurnal expression rhythm of *FvCO* in the SD *F. vesca* and Hawaii-4**.

## References

[B1] Abe M. Kobayashi Y. Yamamoto S. Daimon Y. Yamaguchi A. Ikeda Y. (2005). FD, a bZIP protein mediating signals from the floral pathway integrator FT at the shoot apex. Science 309, 1052–1056 10.1126/science.1115983 16099979

[B2] Bagnall D. J. (1996). Blue-light promotion of flowering is absent in hy4 mutants of Arabidopsis. Planta 200, 278–280 8904811 10.1007/BF00208319

[B3] Böhlenius H. Huang T. Charbonnel-Campaa L. Brunner A. M. Jansson S. Strauss S. H. (2006). *CO/FT* regulatory module controls timing of flowering and seasonal growth cessation in trees. Science 312, 1040–1043 10.1126/science.1126038 16675663

[B4] Bradford E. Hancock J. F. Warner R. M. (2010). Interactions of temperature and photoperiod determine expression of repeat flowering in strawberry. J. Amer. Soc. Hort. Sci. 135, 102–107

[B5] Brambilla V. Fornara F. (2013). Molecular control of flowering in response to day length in rice. J. Integr. Plant Biol. 55, 410–418 10.1111/jipb.12033 23331542

[B6] Brown J. A. M. Klein W. H. (1971). Photomorphogenesis in *Arabidopsis thaliana* (L) Heynh - threshold intensities and blue-far-red synergism in floral induction. Plant Physiol. 47, 393–399 10.1104/pp.47.3.393 16657629 PMC365876

[B7] Brown T. Wareing P. F. (1965). The genetical control of the everbearing habit and three other characters in varieties of Fragaria vesca. Euphytica 14, 97–112

[B8] Cerdán P. D. Chory J. (2003). Regulation of flowering time by light quality. Nature 423, 881–885 10.1038/nature01636 12815435

[B9] Corbesier L. Vincent C. Jang S. Fornara F. Fan Q. Searle I. (2007). FT protein movement contributes to long-distance signaling in floral induction of *Arabidopsis*. Science 316, 1030–1033 10.1126/science.1141752 17446353

[B10] Cremer F. Coupland G. (2003). Distinct photoperiodic responses are conferred by the same genetic pathway in *Arabidopsis* and in rice. Trends Plant Sci. 8, 405–407 10.1016/S1360-1385(03)00192-4 13678904

[B11] Devlin P. Patel S. R. Whitelam G. C. (1998). Phytochrome E influences internode elongation and flowering time in Arabidopsis. Plant Cell 10, 1479–1487 10.1105/tpc.10.9.1479 9724694 PMC144080

[B12] Devlin P. Robson P. R. H. Patel S. R. Goosey L. Sharrock R. A. Whitelam G. C. (1999). Phytochrome D acts in the shade-avoidance syndrome in *Arabidopsis* by controlling elongation growth and flowering time. Plant Physiol. 119, 909–915 10.1104/pp.119.3.909 10069829 PMC32105

[B13] Downs R. Thomas J. (1982). Phytochrome regulation of flowering in the long-day plant, *Hyoscyamus niger* *Plant Physiol* 70, 898–900 10.1104/pp.70.3.898 16662596 PMC1065791

[B14] Griffiths S. Dunford R. P. Coupland G. Laurie D. A. (2003). The evolution of *CONSTANS*-like gene families in barley, rice, and Arabidopsis. Plant Physiol. 131, 1855–1867 10.1104/pp.102.016188 12692345 PMC166942

[B15] Guo H. Yang H. Mockler T. C. Lin C. (1998). Regulation of flowering time by *Arabidopsis* photoreceptors. Science 279, 1360–1363 10.1126/science.279.5355.1360 9478898

[B16] Hayama R. Coupland G. (2004). The molecular basis of diversity in the photoperiodic flowering responses of Arabidopsis and rice. Plant Physiol. 135, 2677–2684 10.1104/pp.104.042614 15208414 PMC514104

[B17] Hayama R. Yokoi S. Tamaki S. Yano M. Shimamoto K. (2003). Adaptation of photoperiodic control pathways produces short-day flowering in rice. Nature 422, 719–722 10.1038/nature01549 12700762

[B18] Heide O. M. (1977). Photoperiod and temperature interactions in growth and flowering of strawberry. Physiol. Plantarum 40, 21–26 10.1111/j.1399-3054.1977.tb01486.x

[B19] Heide O. M. Sønsteby A. (2007). Interactions of temperature and photoperiod in the control of flowering of latitudinal and altitudinal populations of wild strawberry (*Fragaria vesca*). Physiol. Plant. 130, 280–289 10.1111/j.1399-3054.2007.00906.x

[B20] Higuchi Y. Narumi T. Oda A. Nakano Y. Sumimoto K. Fukai S. (2013). The gated induction system of a systemic floral inhibitor, antiflorigen, determines obligate short-day flowering in chrysanthemums. Proc. Natl. Acad. Sci. U.S.A. 110, 17137–17142 10.1073/pnas.1307617110 24082137 PMC3801008

[B21] Hirakawa H. Shirasawa K. Kosugi S. Tashiro K. Nakayama S. Yamada M. (2013). Dissection of the octoploid strawberry genome by deep sequencing of the genomes of *Fragaria species*. DNA Res. 21, 169–181 10.1093/dnares/dst049 24282021 PMC3989489

[B22] Holland R. W. K. Vince D. (1971). Floral initiation in *Lolium temulentum* L.: the role of phytochrome in responses to red and far-red light. Planta 98, 232–243 24493394 10.1007/BF00387068

[B23] Hsu C. Adams J. P. Kim H. No K. Ma C. Strauss S. H. (2011). *FLOWERING LOCUS T* duplication coordinates reproductive and vegetative growth in perennial poplar. Proc. Natl. Acad. Sci. U.S.A. 108, 10756–10761 10.1073/pnas.1104713108 21653885 PMC3127867

[B23a] Hsu C. Adams J. P. No K. Liang H. Meilan R. Pechanova O. (2012). Overexpression of constans homologs CO1 and CO2 fails to alter normal reproductive onset and fall bud set in woody perennial poplar. PLoS ONE 7:e45448 10.1371/journal.pone.0045448 23029015 PMC3446887

[B24] Hu W. Franklin K. A. Sharrock R. A. Jones M. A. Harmer S. L. Lagarias J. C. (2013). Unanticipated regulatory roles for *Arabidopsis* phytochromes revealed by null mutant analysis. Proc. Natl. Acad. Sci. U.S.A. 110, 1542–1547 10.1073/pnas.1221738110 23302690 PMC3557068

[B25] Imaizumi T. Tran H. Swartz T. Briggs W. Kay S. (2003). FKF1 is essential for photoperiodic-specific light signalling in *Arabidopsis*. Nature 426, 302–306 10.1038/nature02090 14628054

[B26] Ishikawa R. Aoki M. Kurotani K. Yokoi S. Shinomura T. Takano M. (2011). Phytochrome B regulates *Heading date 1 (Hd1)*-mediated expression of rice florigen *Hd3a* and critical day length in rice. Mol. Genet. Genomics 285, 461–470 10.1007/s00438-011-0621-4 21512732

[B27] Iwata H. Gaston A. Remay A. Thouroude T. Jeauffre J. Kawamura K. (2012). The *TFL1* homologue *KSN* is a regulator of continuous flowering in rose and strawberry. Plant J. 69, 116–125 10.1111/j.1365-313X.2011.04776.x 21895811

[B28] Jaeger K. E. Wigge P. A. (2007). FT protein acts as a long-range signal in *Arabidopsis*. Curr. Opin. Plant Biol. 17, 1050–1054 10.1016/j.cub.2007.05.008 17540569

[B29] Johnson E. Bradley M. Harberd N. P. Whitelam G. C. (1994). Photoresponses of light-grown *phyA* mutants of *Arabidopsis*. Plant Physiol. 105, 141–149 10.1104/pp.105.1.141 12232194 PMC159339

[B30] Karimi M. Inzé D. Depicker A. (2002). GATEWAY vectors for Agrobacterium –mediated plant transformation. Trends Plant Sci. 7, 193–195 10.1016/S1360-1385(02)02251-3 11992820

[B31] Kim S. Y. Yu X. Michaels S. D. (2008). Regulation of *CONSTANS* and *FLOWERING LOCUS T* expression in response to changing light quality. Plant Physiol. 148, 269–279 10.1104/pp.108.122606 18667727 PMC2528114

[B32] Kojima S. Takahashi Y. Kobayashi Y. Monna L. Sasaki T. Araki T. (2002). *Hd3a*, a rice ortholog of the Arabidopsis *FT* gene, promotes transition to flowering downstream of *Hd1* under short-day conditions. Plant Cell Physiol. 43, 1096–1105 10.1093/pcp/pcf156 12407188

[B33] Koskela E. A. Mouhu K. Albani M. C. Kurokura T. Rantanen M. Sargent D. J. (2012). Mutation in *TERMINAL FLOWER1* reverses the photoperiodic requirement for flowering in the wild strawberry *Fragaria vesca*. Plant Physiol. 159, 1043–1054 10.1104/pp.112.196659 22566495 PMC3387692

[B34] Kurokura T. Mimida N. Battey N. H. Hytönen T. (2013). The regulation of seasonal flowering in the Rosaceae. J. Exp. Bot. 64, 4131–4141 10.1093/jxb/ert233 23929655

[B35] Lagercrantz U. (2009). At the end of the day: a common molecular mechanism for photoperiod responses in plants? J. Exp. Bot. 60, 2501–2515 10.1093/jxb/erp139 19414498

[B36] Ledger S. Strayer C. Ashton F. Kay S. A. Putterill J. (2001). Analysis of the function of two circadian-regulated *CONSTANS-LIKE* genes. Plant J. 26, 15–22 10.1046/j.1365-313x.2001.01003.x 11359606

[B37] Martinez-Zapater J. M. Somerville C. R. (1990). Effect of light quality and vernalization on late-flowering mutants of *Arabidopsis thaliana*. Plant Physiol. 92, 770–776 10.1104/pp.92.3.770 16667348 PMC1062367

[B38] Meijer G. (1959). Photomorphogenesis in different spectral regions, in Photoperiodism and Related Phenomena in Plants and Animals, ed Withrow R. B. (Washington, DC: American association for the advancement of science), 101–109

[B39] Mockler T. Yang H. Yu X. Parikh D. Cheng Y. Dolan S. (2003). Regulation of photoperiodic flowering by *Arabidopsis* photoreceptors. Proc. Natl. Acad. Sci. U.S.A. 100, 2140–2145 10.1073/pnas.0437826100 12578985 PMC149972

[B40] Mockler T. C. Guo H. W. Yang H. Y. Duong H. Lin C. T. (1999). Antagonistic actions of *Arabidopsis* cryptochromes and phytochrome B in the regulation of floral induction. Development 126, 2073–2082 10207133 10.1242/dev.126.10.2073

[B41] Monte D. Somerville S. (2002). Pine tree method for isolation of plant RNA, in DNA Microarrays: A Molecular Cloning Manual, eds Bowtel D. Sambrook J. (New York, NY: Cold Spring Harbour Laboratory Press), 124–126

[B42] Moon J. Lee H. Kim M. Lee I. (2005). Analysis of flowering pathway integrators in *Arabidopsis*. Plant Cell Physiol. 46, 292–299 10.1093/pcp/pci024 15695467

[B43] Mouhu K. Hytonen T. Folta K. Rantanen M. Paulin L. Auvinen P. (2009). Identification of flowering genes in strawberry, a perennial SD plant. BMC Plant Biol. 99:122 10.1186/1471-2229-9-122 19785732 PMC2761920

[B44] Mouhu K. Kurokura T. Koskela E. A. Albert V. A. Elomaa P. Hytonen T. (2013). The *Fragaria vesca* homolog of SUPPRESSOR OF OVEREXPRESSION OF CONSTANS1 represses flowering and promotes vegetative growth. Plant Cell 25, 3296–3310 10.1105/tpc.113.115055 24038650 PMC3809533

[B45] Oosumi T. Hope A. Gruszewski H. A. Blischak L. A. Baxter A. J. Wadl P. A. (2006). High-efficiency transformation of the diploid strawberry (*Fragaria vesca*) for functional genomics. Planta 223, 1219–1230 10.1007/s00425-005-0170-3 16320068

[B46] Pin P. A. Benlloch R. Bonnet D. Wremerth-Weich E. Kraft T. Gielen J. J. L. (2010). An antagonistic pair of *FT* homologs mediates the control of flowering time in sugar beet. Science 330, 1397–1400 10.1126/science.1197004 21127254

[B47] Pin P. A. Nilsson O. (2012). The multifaceted roles of FLOWERING LOCUS T in plant development. Plant Cell Environ. 35, 1742–1755 10.1111/j.1365-3040.2012.02558.x 22697796

[B48] Reed J. W. Nagatani A. Elich T. D. Fagan M. Chory J. (1994). Phytochrome A and Phytochrome B have overlapping but distinct functions in *arabidopsis* development. Plant Physiol. 104, 1139–1149 10.1104/pp.104.4.1139 12232154 PMC159274

[B49] Sawa M. Nusinow D. A. Kay S. A. Imaizumi T. (2007). FKF1 and GIGANTEA complex formation is required for day-length measurement in Arabidopsis. Science 318, 261–265 10.1126/science.1146994 17872410 PMC3709017

[B50] Shulaev V. Sargent D. J. Crowhurst R. N. Mockler T. C. Folkerts O. Delcher A. L. (2011). The genome of woodland strawberry (*Fragaria vesca*). Nat. Genet. 43, 109–116 10.1038/ng.740 21186353 PMC3326587

[B51] Song Y. H. Smith R. W. To B. J. Millar A. J. Imaizumi T. (2012). FKF1 conveys timing information for CONSTANS stabilization in photoperiodic flowering. Science 336, 1045–1049 10.1126/science.1219644 22628657 PMC3737243

[B52] Sønsteby A. Heide O. M. (2007). Long-day control of flowering in everbearing strawberries. J. Hort. Sci. Biotech. 82, 875–884 19785732

[B53] Sønsteby A. Heide O. M. (2008). Long-day rather than autonomous control of flowering in the diploid everbearing strawberry Fragaria vesca ssp semperflorens. J. Hort. Sci. Biotech. 83, 360–366

[B54] Stewart P. J. (2007). Molecular Characterization of Photoperiodic Flowering in Strawberry (Fragaria sp.). Ph.D. thesis, University of Florida

[B55] Strasser B. Sánchez-Lamas M. Yanovsky M. J. Casal J. J. Cerdán P. D. (2010). *Arabidopsis thaliana* life without phytochromes. Proc. Natl. Acad. Sci. U.S.A. 107, 4776–4781 10.1073/pnas.0910446107 20176939 PMC2842051

[B56] Suarez-Lopez P. Wheatley K. Robson F. Onouchi H. Valverde F. Coupland G. (2001). *CONSTANS* mediates between the circadian clock and the control of flowering in *Arabidopsis*. Nature 410, 1116–1120 10.1038/35074138 11323677

[B57] Takano M. Inagaki N. Xie X. Kiyota S. Baba-Kasai A. Tanabata T. (2009). Phytochromes are the sole photoreceptors for perceiving red/far-red light in rice. Proc. Natl. Acad. Sci. U.S.A. 106, 14705–14710 10.1073/pnas.0907378106 19706555 PMC2732857

[B58] Tamaki S. Matsuo S. Wong H. L. Yokoi S. Shimamoto K. (2007). Hd3a protein is a mobile flowering signal in rice. Science 316, 1033–1036 10.1126/science.1141753 17446351

[B59] Taoka K. Ohki I. Tsuji H. Furuita K. Hayashi K. Yanase T. (2011). 14-3-3 proteins act as intracellular receptors for rice Hd3a florigen. Nature 476, 332–335 10.1038/nature10272 21804566

[B60] Torti S. Fornara F. Vincent C. Andrés F. Nordström K. Göbel U. (2012). Analysis of the *Arabidopsis* shoot meristem transcriptome during floral transition identifies distinct regulatory patterns and a leucine-rich repeat protein that promotes flowering. Plant Cell 24, 444–462 10.1105/tpc.111.092791 22319055 PMC3315226

[B61] Tsuji H. Taoka K. Shimamoto K. (2011). Regulation of flowering in rice: two florigen genes, a complex gene network, and natural variation. Curr. Opin. Plant Biol. 14, 45–52 10.1016/j.pbi.2010.08.016 20864385

[B62] Valverde F. Mouradov A. Soppe W. Ravenscroft D. Samach A. Coupland G. (2004). Photoreceptor regulation of CONSTANS protein in photoperiodic flowering. Science 303, 1003–1006 10.1126/science.1091761 14963328

[B63] Vince-Prue D. Guttridge C. G. (1973). Floral initiation in strawberry—spectral evidence for regulation of flowering by long-day inhibition. Planta 110, 165–172 24474344 10.1007/BF00384839

[B64] Wigge P. A. Kim M. C. Jaeger K. E. Busch W. Schmid M. Lohmann J. U. (2005). Integration of spatial and temporal information during floral induction in *Arabidopsis*. Science 309, 1056–1059 10.1126/science.1114358 16099980

[B65] Wollenberg A. C. Strasser B. Cerdan P. D. Amasino R. M. (2008). Acceleration of flowering during shade avoidance in Arabidopsis alters the balance between *FLOWERING LOCUS C*-mediated repression and photoperiodic induction of flowering. Plant Physiol. 148, 1681–1694 10.1104/pp.108.125468 18790998 PMC2577263

[B66] Yanovsky M. J. Kay S. A. (2002). Molecular basis of seasonal time measurement in *Arabidopsis*. Nature 419, 308–312 10.1038/nature00996 12239570

[B67] Yoo S. K. Chung K. S. Kim J. Lee J. H. Hong S. M. Yoo S. J. (2005). *CONSTANS* activates *SUPPRESSOR OF OVEREXPRESSION OF CONSTANS 1* through *FLOWERING LOCUS T* to promote flowering in Arabidopsis. Plant Physiol. 139, 770–778 10.1104/pp.105.066928 16183837 PMC1255994

