# Supplementary material for: Light quality regulates flowering in FvFT1/FvTFL1 dependent manner in the woodland strawberry Fragaria vesca
Source: Front Plant Sci. 2014 Jun 11;5:271. doi: 10.3389/fpls.2014.00271 (PMC4052200; doi:10.3389/fpls.2014.00271)
Supplement: Supplementary Table 1 — qPCR primers used in this study. [file DataSheet1.PDF]

Supplementary Material

Light quality regulates flowering in *FvFT1/FvTFL1* dependent manner in the woodland strawberry *Fragaria vesca*

Marja Rantanen[1], Takeshi Kurokura[1], Katriina Mouhu[1], Paulo Pinho[2], Eino Tetri[2], Liisa Halonen[2], Pauliina Palonen[1], Paula Elomaa[1] and Timo Hytönen[1]*

[1] Department of Agricultural Sciences, University of Helsinki, Helsinki, Finland
[2] Department of Electrical Engineering and Automation, Aalto University, Espoo, Finland

*** Correspondence:** Timo Hytönen, Department of Agricultural Sciences, University of Helsinki, P.O.Box 27, FI-00014 Helsinki, Finland.
timo.hytonen@helsinki.fi

1. Supplementary Figures and Tables

Table 1. qPCR primers used in this study
Figure 1. qPCR program used in this study
Figure 2. Stability of the normalization gene *FvMSI1*
Figure 3. Flowering time and *FvFT1* expression in Hawaii-4 at 22°C
Figure 4. The down-regulation of *FvSOC1* in the RNAi-lines
Figure 5. Diurnal expression rhythm of *FvCO* in the SD *F. vesca* and Hawaii-4

1.1. Supplementary Tables

Supplementary Table 1. qPCR primers used in this study.

Gene	Forward primer sequence	Reverse primer sequence	Primer efficiency
FvMSI1	tccccacacctttgattgcca	acaccatcagtctcctgccaag	2.044
FvSOC1	acttgctgggttcattttcc	gagctttcctctgggagaga	1.956
FvTFL1	ctggcaccacagatgctaca	aacggcagcaacaggaac	1.994
FvAP1	agctcaggaggttcatgactg	taaggtcgagctggttcctc	1.938
FvFUL1	gcagtgcatgaatccctttc	gctggtgattttggagcttg	1.986
FvFT1	caatctcttggccgaaaact	tgagctcaaaccttcccaag	1.969
FvCO	gacatccactccgccaac	gtggaccccaccactatctg	1.996

1.2. Supplementary Figures

Programs

Program Name	Pre-incubation		
Cycles	1	Analysis Mode	None

Target (°C)	Acquisition Mode	Hold (hh:mm:ss)	Ramp Rate (°C/s)	Acquisitions (per °C)	Sec Target (°C)	Step size (°C)	Step Delay (cycles)
95	None	00:10:00	4,80		0	0	0

Program Name	Amplification/Quantification		
Cycles	45	Analysis Mode	Quantification

Target (°C)	Acquisition Mode	Hold (hh:mm:ss)	Ramp Rate (°C/s)	Acquisitions (per °C)	Sec Target (°C)	Step size (°C)	Step Delay (cycles)
95	None	00:00:10	4,80		0	0	0
59	None	00:00:10	2,50		0	0	0
72	Single	00:00:10	4,80		0	0	0

Program Name	Melting Curve Analysis		
Cycles	1	Analysis Mode	Melting Curves

Target (°C)	Acquisition Mode	Hold (hh:mm:ss)	Ramp Rate (°C/s)	Acquisitions (per °C)	Sec Target (°C)	Step size (°C)	Step Delay (cycles)
95	None	00:00:05	4,80		0	0	0
65	None	00:01:00	2,50		0	0	0
97	Continuous		0,11	5	0	0	0

Program Name	Cooling		
Cycles	1	Analysis Mode	None

Target (°C)	Acquisition Mode	Hold (hh:mm:ss)	Ramp Rate (°C/s)	Acquisitions (per °C)	Sec Target (°C)	Step size (°C)	Step Delay (cycles)
40	None	00:00:30	2,50		0	0	0

Supplementary Figure 1. qPCR program (LightCycler 480, Roche) used in this study.

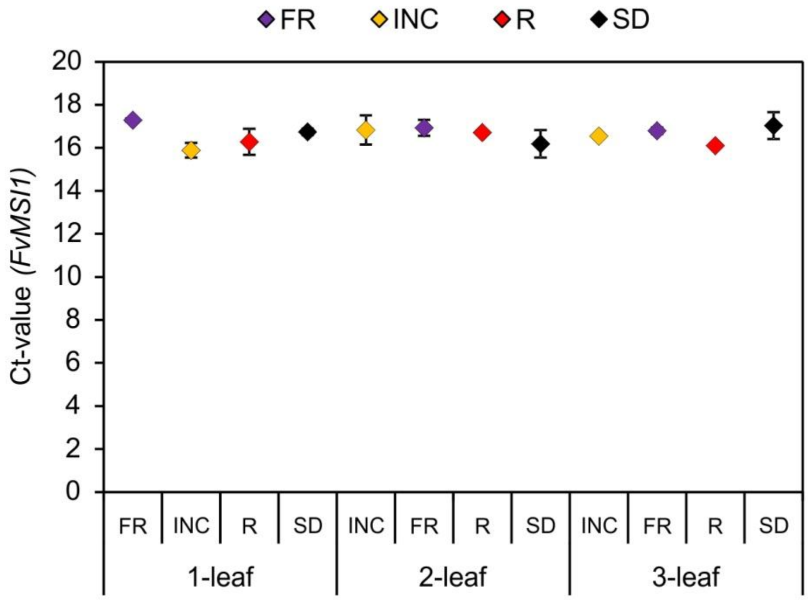

Supplementary Figure 2. Stability of the normalization gene *FvMSI1* used in qPCR. Roche LightCycler 480 Cp values for *FvMSI1* in the *F. vesca* (Hawaii-4) shoot apex samples collected under different light quality treatments at different growth stages. Plants were subjected to 12-h short-day (SD) or 12-h SD plus 6-h low intensity (8 µmol $m^{-2}s^{-1}$) end-of-day treatment with far-red, incandescent, or red light (FR, INC, and R, respectively). Values are means of two biological replicates ± SD. If invisible, SD is smaller than the marker.

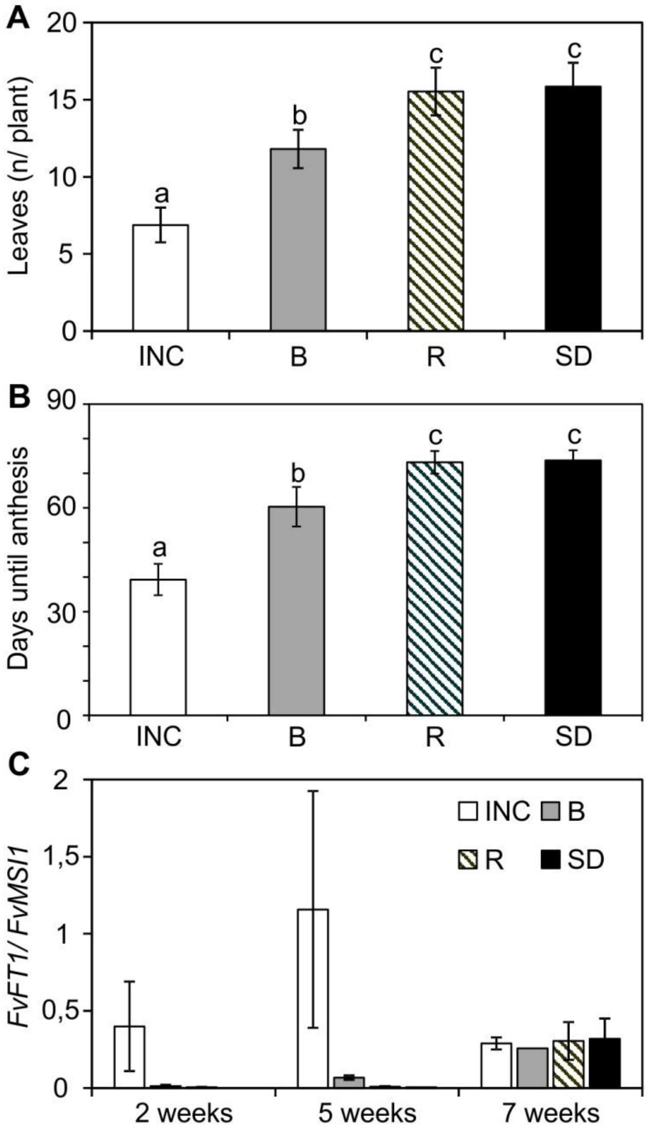

Supplementary Figure 3. The effect of light quality on flowering time and *FvFT1* expression in perpetual flowering *F. vesca* accession Hawaii-4 (H4) at 22°C. (A) Flowering time of the seedlings of H4 indicated as the number of leaves in the primary leaf rosette before the terminal inflorescence (B) Flowering time of H4 seedlings indicated as days from the beginning of the treatment. (n = 30–33). Flowering results were subjected to one-way ANOVA (t-test), $p < 0.001$ for the treatment. Different lower-case letters indicate significant difference between the treatments in Tukey's pairwise test, $\alpha = 0.05$. (C) The expression of *FvFT1* in the leaves of H4. Plants with 2–3 open leaves were subjected to 12-h short-day (SD) or 12-h SD plus 6-h low intensity (20 µmol $m^{-2}s^{-1}$) end-of-day treatment with incandescent, blue or red light (INC, B and R, respectively) at 22°C for six weeks. For gene expression data, three biological replicates were analyzed by real-time PCR. Leaf samples were collected 16 hours after dawn. Results are mean ± SD.

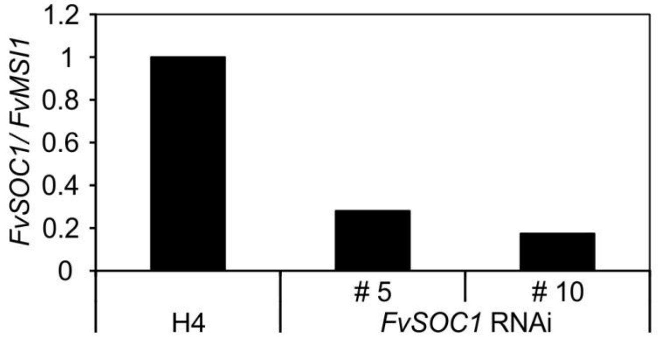

Supplementary Figure 4. The down-regulation of *FvSOC1* in *FvSOC1* RNAi lines #5 and #10 compared to H4. Plants were grown under LD conditions.

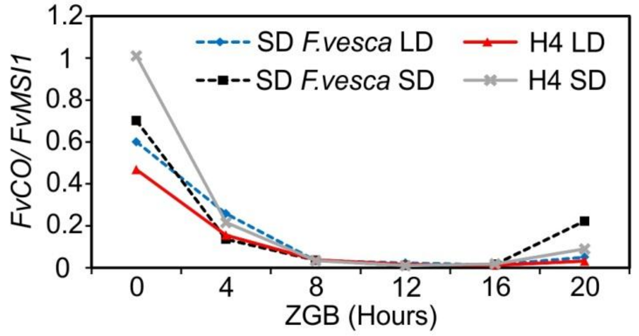

Supplementary Figure 5. Diurnal expression rhythm of *FvCO* under short day (SD) and long day (LD) conditions in the SD *F. vesca* and perpetual flowering accession Hawaii-4 (H4). Values are means of three biological replicates.
